# Better than one: a synthetic community of Gram-positive bacteria protects pepper plants from aphid infestation through *de novo* volatile production

**DOI:** 10.3389/fpls.2025.1589266

**Published:** 2025-05-29

**Authors:** Sang-Moo Lee, Hyeonu Yang, Hyun Gi Kong, Myoungjoo Riu, Choong-Min Ryu

**Affiliations:** ^1^ Molecular Phytobacteriology Laboratory, Infectious Disease Research Center, Korea Research Institute of Bioscience and Biotechnology (KRIBB), Daejeon, Republic of Korea; ^2^ Department of Biosystems and Bioengineering, Korea Research Institute of Bioscience and Biotechnology (KRIBB) School of Biotechnology, University of Science and Technology, Daejeon, Republic of Korea; ^3^ Institute of Agricultural Life Sciences, Dong-A University, Busan, Republic of Korea; ^4^ Department of Plant Medicine, College of Agriculture, Life and Environment Sciences, Chungbuk National University, Cheongju, Republic of Korea

**Keywords:** rhizobacteria, plant immunity, biological control, volatile, aphid, pepper

## Abstract

Soil microbes offer various benefits to plants, including induced systemic resistance and growth promotion, with some functioning as biocontrol agents. Although the role of microbial consortium in microbiota function was recently elucidated, the production of a specific determinant through microbial cooperation for plant protection against insect infestation has not been demonstrated to date. Here, we report that a synthetic community (SynCom) comprising four Gram-positive bacteria could protect pepper plants from aphid infestation under greenhouse and field conditions. Headspace solid-phase microextraction-gas chromatography mass spectrometry analysis of the determinants produced by the four bacteria during co-cultivation led to the *de novo* detection of a volatile compound, 1-nonanol. Drench application of 1 mM 1-nonanol reduced aphid infestation. Taken together, our results suggest that SynCom and its volatile compound can effectively attenuate insect infestation. This is the first case study demonstrating how a volatile compound synthesized in the rhizosphere soil by bacteria protects plants against invasion by a sucking insect pest.

## 1 Introduction

In nature, microbial colonization in the rhizosphere soil facilitates plant adaptation to various environments by promoting plant growth (Zablotowicz et al., 1991; Hayat et al., 2010), enhancing abiotic stress tolerance (Yang et al., 2009), and defending plants against pathogen invasion (Kloepper et al., 2004; Berendsen et al., 2012; Kwak et al., 2018; Lee et al., 2021). Certain rhizobacteria act as bioprotectants and biofertilizers by protecting plants from diseases and enhancing crop yield (Kloepper et al., 2004; Berg, 2009). Traditionally, beneficial bacterial species have been applied individually to agricultural fields; however, their effectiveness as bioprotectants and biostimulants has often been unstable. This instability under field conditions is attributed to the complex microbial interactions within natural communities (Martins et al., 2023). To address this issue, the co-inoculation of multiple bacterial species that mimic the natural microbiota, known as synthetic communities (SynComs), has recently been proposed (Vorholt et al., 2017; Durán et al., 2018; Martins et al., 2023).

The construction of SynComs can be achieved through the random selection of various natural isolates or through the integrated prediction of microbiota and plant phenotypes based on *in silico* approaches, such as metagenome sequencing (Martins et al., 2023). Accounting for microbial interactions is essential for SynCom construction. Microbial interactions among SynCom members, as well as those between SynCom members and native microbiota, play a crucial role in the overall functioning and stability of SynComs in nature (Lee et al., 2024). Recently, in addition to identifying keystone taxa important for the functionality and stability of SynComs, identifying rare taxa with relatively low abundance has been recognized as an essential consideration for ensuring the full activity of SynCom members (Jousset et al., 2017; Carlström et al., 2019; Lee et al., 2021; Xiong et al., 2021; Kim et al., 2023; Lee et al., 2024). In recent studies, diverse SynComs were constructed to investigate plant-microbiome interactions for causality determination, yet the chemical determinants underlying SynCom functionality remain largely unknown.

Microorganisms do not exist individually in nature, rather in multi-species communities, where biological diversity leads to chemical diversity (Martins et al., 2023). The chemicals produced by microbes include volatile and non-volatile secondary metabolites. A key feature of volatiles is their ability to travel long distances through air and soil, making them effective mediators of microbe-microbe or plant-microbe interactions (Ryu et al., 2003, 2004; Cernava et al., 2015; Silva Dias et al., 2021; Weisskopf et al., 2021). Bacterial volatiles, including 2,3-butanediol and acetoin, are known to promote plant growth and activate plant immunity (Garbeva and Weisskopf, 2020; Silva Dias et al., 2021; Gfeller et al., 2022; Almeida et al., 2023; Singh et al., 2024; Belt et al., 2025). For instance, 2,3-butanediol released by *Bacillus velezensis* strain GB03 (previously *Bacillus amyloliquefaciens*) was first reported to enhance plant growth and immunity in *Arabidopsis thaliana* (Ryu et al., 2003, 2004). Subsequent studies reported that bacterial volatile compounds protect host plants against microbial pathogens, including viruses, bacteria, and fungi, as well as insect pests (Cortes-Barco et al., 2010; Song and Ryu, 2013; Kong et al., 2018; Gfeller et al., 2022; Jung et al., 2023; Singh et al., 2024; Belt et al., 2025). Previously, most studies investigating the beneficial effects of volatiles focused on single bacterial species. Consequently, little is known about the biosynthesis of volatile compounds by SynCom for crop protection. However, a recent study reported differences in volatile composition between bacterial monocultures and mixed cultures (Schulz-Bohm et al., 2015). In addition, loss of the unique functionality of microbial volatiles was observed when specific taxa were absent from a microbial community (Hol et al., 2015). Thus, these data suggest the potential for the biosynthesis of specific volatile(s) that determine the functionality of SynCom.

Previously, based on the microbiome analysis of rhizosphere soil in tomato (*Solanum lycopersicum* L.) fields, we constructed a protective SynCom comprising four Gram-positive bacteria, namely, *Brevibacterium frigoritolerans* HRS1, *Bacillus niacini* HRS2, *Solibacillus silvestris* HRS3, and *Bacillus luciferensis* HRS4 (Lee et al., 2021). This SynCom efficiently reduced the occurrence of bacterial wilt disease, caused by *Ralstonia pseudosolanacearum*, in tomato by activating plant immunity (Lee et al., 2021). A complete combination of SynCom with keystone taxa and rare taxa can induce the full activation of plant immunity in tomato. However, the ability of SynCom to control naturally-occurring plant diseases and insect pests and the SynCom-derived determinants involved in this process remain largely unknown.

The aim of the present study is to investigate the biocontrol activity of the SynCom under field conditions. To validate this, we applied the SynCom to the root system of pepper plants instead of tomato plants, as tomatoes are generally cultivated in greenhouse conditions in S. Korea. Drenching application of SynCom effectively protects pepper (*Capsicum annum* L.) plants against aphid (*Myzus persicae* L.) infestation, even under field conditions. Compared with individual bacterium inoculations, the SynCom treatment showed higher biocontrol activity against aphid infestation in the field. The four bacterial species when used together as the SynCom specifically produced the volatile compound 1-nonanol. Thus, our data highlight the role of a specific combination of bacterial species (HRS1 + 2+3 + 4) in the production of a unique metabolite for host plant protection.

## 2 Materials and methods

### 2.1 Plant materials

Pepper (*Capsicum annum* L. cv. Bulkala) seeds were sown on autoclaved soilless potting medium (Punong, Co. Ltd., Gyeongju, South Korea), containing zeolite, perlite, colored dust, and lime (pH = 4.5–7.5), in a 50-hole plastic tray (28 cm × 54 cm × 5 cm). After 7 days, pepper seedlings were transplanted in new round pots (diameter = 10 cm, height = 8.5 cm) containing autoclaved soilless potting medium. Pepper plants were grown in an environmentally controlled growth room at 25°C under fluorescent lights (approximately 7,000 lux light intensity) and 12 h light/12 h dark cycle.

### 2.2 Evaluation of SynCom and 1-nonanol against aphid infestation in the greenhouse

Four different Gram-positive bacteria (*Brevibacterium frigoritolerans* HRS1, *Bacillus niacini* HRS2, *Solibacillus silvestris* HRS3, and *Bacillus luciferensis* HRS4) were cultured as described previously. Briefly, four bacterial isolates were cultured on Tryptic Soy Agar (TSA, Difco Laboratories, Detroit, MI, USA) medium at 30 °C for 1 day and suspended in sterile distilled water (OD_600_ = 1.0). To prepare the SynCom suspension, the four strains were initially prepared at a higher concentration and then mixed in calculated volumes to achieve a final OD_600_ of 1.0 for each bacterium. To conduct the greenhouse experiment, 10 mL of the SynCom suspension was drenched into the root system of 3-week-old pepper plants twice a week, and the number of aphids on the aerial parts of each plant was counted at 0, 7, and 14 days after inoculation. Then, 10 mL of 1-nonanol at four different concentrations (1 μM, 10 μM, 100 μM, and 1 mM) was drenched into the root system of 3-week-old pepper plants. Drench application of 10 mL of 0.5 mM of benzothiadiazole (BTH) and sterile distilled water (SDW) served as positive and negative controls, respectively, and the number of aphids on the aerial parts of each plant was counted at 3, 4, 5, 6, and 7 days after inoculation.

Aphid (*Myzus persicae* L.) adults were obtained from the Department of Agro-Food Safety and Crop Protection, National Institute of Agricultural Sciences, Rural Development Administration, South Korea. Aphids were reared in a miniature plastic box (70 cm wide × 70 cm long × 80 cm tall). Then, 10 aphids were transferred onto the apex of freshly grown pepper plants using a small paint brush one week after the SynCom or 1-nonanol treatment.

### 2.3 Field trials

Field trials were conducted at Nonsan, Chungcheongnam-do, South Korea (36.23577°N, 127.18946°E). All necessary permits were obtained from landowners prior to the commencement of field trials. To evaluate the biocontrol activity of SynCom under typical field conditions, irrigation and fertilizer treatments were applied uniformly across all plots, including the negative control, by the farm owner, according to the environmental conditions at the experimental site throughout the entire trial period. Before transplanting, the furrows were covered with black polyethylene film to prevent weed growth. Pepper seedlings were planted 30 cm apart. After 30 days of growth, seedlings were irrigated with bacterial suspensions (OD_600_ = 1.0), 0.5 mM BTH, or SDW (100 mL per seedling) every 10 days, for a total of three times per month. To prepare the SynCom suspension (a mixture of HRS1, HRS2, HRS3, and HRS4), suspensions of all four strains were mixed, and the final OD_600_ of each bacteria was adjusted to 1.0. Each treatment was applied to four blocks, in a randomized block design (n = nine plants per treatment). For GC–MS analysis, we employed an Agilent 7890A series gas chromatograph (Agilent Technologies, Santa Clara, CA, United States).

To inoculate pepper plants, *Xanthomonas axonopodis* pv. *vesicatoria* (*Xav*) was cultured overnight at 28 °C in LB medium. At 2 weeks after inoculation, 500 μL of *Xav* (OD_600_ = 0.01) culture was pressure-infiltrated into the abaxial surface of pepper leaves using a needleless syringe. Seven days after inoculation, the severity of disease caused by *Xav* was recorded on a 0–5 scale, where 0 = no symptom; 1 = mild chlorosis; 2 = chlorosis; 3 = severe chlorosis and mild necrosis; 4 = necrosis; and 5 = necrosis with cell death (Kim et al., 2022). Each treatment was applied to four blocks, with five plants per treatment, in a randomized block design.

### 2.4 Evaluation of aphid infestation in pepper under field conditions

To evaluate the biological control activity of SynCom under field conditions, we measured the severity of aphid infestation at 4 weeks after treatment at the Nonsan field. The severity of aphid infestation was recorded on a 0–5 scale based on the distribution of aphids in the aboveground parts of the pepper: 0 = no infestation; 1 <25% of the pepper plant infested; 2 <50% of the pepper plant infested; 3 <75% of the pepper plant infested; 4 <100% of the pepper plant infested; and 5 = whole plant infested. Each treatment was replicated four or five times in a randomized block design (n = 36 plants per treatment).

### 2.5 Pepper fruit yield measurement

Fruit fresh weight per plant and number of pepper fruits per 20 plants in a row were measured at 16 weeks after transplanting, with four replications. Only red-colored fruits were harvested for market value. Total yield (g/plant) was estimated per treatment, and the total fruit weight per plant was calculated. In addition, the number of fruits per plant was recorded at each harvest, and the total harvest was then calculated as the number of fruits per plant.

### 2.6 Detection of *de novo* volatile compound production

Volatile compounds produced by the SynCom were identified by headspace solid-phase microextraction-gas chromatography mass spectrometry (HS-SPME-GC-MS). Briefly, 50 μL of each bacterial suspensions was inoculated on the TSA medium and cultured in 20-mL SPME vials at 30°C for 2 days. Suspension cultures of the four different bacterial species were mixed in different combinations (HRS1 + HRS2, HRS1 + HRS2 + HRS3, HRS1 + HRS2 + HRS4, and HRS1 + HRS2 + HRS3 + HRS4), and the final OD_600_ of each mixed bacterial species, as well as that of monocultures (HRS1, HRS2, HRS3, and HRS4), was adjusted to 1.0. The equipment condition of HS-SPME-GC-MS was modified using the method described previously (Song et al., 2019). Briefly, the fibers were conditioned in the GC injection port prior to use, according to the manufacturer’s instructions. A manual holder was used to handle the fibers. Separation was performed using the following program: initial temperature of 50°C with a 2-min hold, followed by ramping up to 220°C at a rate of 10°C/min with a 2-min hold. The split–splitless injection port was maintained at 280°C for volatile desorption in split mode, with a split ratio of 1:10. Helium was employed as the carrier gas at a constant flow rate of 1.0 mL/min. Volatile organic compounds (VOCs) were separated using a non-polar HP-5MS column (30 m × 0.25 mm × 0.25 µm, Hewlett Packard) with the following program: initial temperature of 40°C with a 3-min hold, followed by ramping up to 220°C at 10°C/min with a 2-min hold. The split–splitless injection port was set to 250°C in splitless mode. The MS parameters were set to the full-scan mode, with a range of 40–500 amu at a scan rate of 0.817 scan/s. The ion source temperature was 250°C, with an ionization energy of 70 eV and a mass transfer line temperature of 300°C. The retention time (tR) and mass spectrum of each VOC was compared with those of authentic standards and of volatiles from the National Institute of Standards and Technology (NIST) reference library. An in-house dedicated mass spectral library, containing the spectra of known compounds, was also used to verify the identity of the detected VOCs.

### 2.7 Expression analysis of defense-related marker genes in pepper leaves

Total RNA was isolated from pepper leaves collected at 8 days post-aphid inoculation, and first-strand cDNA was synthesized as previously described (Song et al., 2017). Quantitative real-time PCR (qRT-PCR) was carried out on the Chromo4 Real-Time PCR System (Bio-Rad, Hercules, CA, USA) using iQ™ SYBR^®^ Green SuperMix (Bio-Rad), 10 pM sequence-specific primers (**Supplementary Table 1**) (Lee et al., 2012; Yi et al., 2013; Kong et al., 2018), and cDNA (template). The reaction conditions were as follows: an initial polymerase activation step at 95°C for 10 min, followed by 40 cycles of denaturation at 95°C for 30 s, annealing at 60°C for 30 s, and extension at 72°C for 30 s. Gene expression levels were calibrated and normalized against the mRNA level of *CaActin*.

### 2.8 Statistical analysis

Data were analyzed using analysis of variance (ANOVA) in JMP 4.0 software (SAS Institute Inc., Cary, NC, USA). Significant treatment effects were determined based on the F-value at a significance level of *p* < 0.05. When a significant F-value was obtained, *post hoc* pairwise comparisons were conducted using Fisher’s protected least significant difference (LSD) or Tukey’s honestly significant difference (HSD) test at *p* < 0.05. For ordinal disease severity measurements related to naturally occurring aphid infestations and *Xav* infections, statistically significant differences were assessed using the nonparametric Kruskal-Wallis test, followed by Dunn’s *post hoc* test for multiple comparisons, implemented in R (http://www.r-project.org/).

## 3 Results

### 3.1 Gram-positive bacterial SynCom reduced aphid infestation in pepper

**Figure 1:**
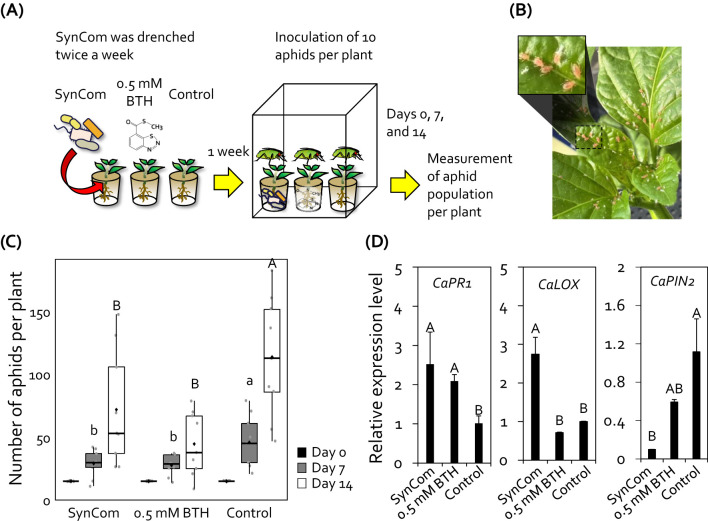
Synthetic community (SynCom) comprising four Gram-positive bacteria reduced aphid infestation in pepper plants. **(A)** Experimental procedure for the aphid control assay. SynCom (10 mL) was drenched into the root system of 3-week-old pepper plants twice a week. One week after the SynCom treatment, each pepper plant was inoculated with 10 aphids. The total population of aphids on the aerial parts of plants were counted at 0, 7, and 14 days post-inoculation. **(B)** Photograph showing aphid infestation on the aboveground parts of pepper plants at 14 days post-inoculation. **(C)** Total number of aphids on SynCom-treated pepper plants. SynCom, mixture of *Brevibacterium frigoritolerans* HRS1, *Bacillus niacini* HRS2, *Solibacillus silvestris* HRS3, *Bacillus luciferensis* HRS4; BTH, 0.5 mM benzothiadiazole (positive control); SDW, sterile distilled water (negative control). Data represent mean ± standard error of the mean (SEM; *n* = 18 replications per treatment). Different letters indicate significant differences between treatments (*P* < 0.05; least significant difference [LSD] test). Diamonds and bolded lines of the boxplot are the average and median of indicated values, respectively. **(D)** Relative expression levels of salicylic acid signaling marker gene (*CaPR*) and jasmonic acid signaling marker gene (*CaLOX* and *CaPIN2*) in the leaves of pepper plants treated with 1-nonanol at 24 hour post-inoculation with aphids. Data represent mean ± SEM. Different letters indicate significant differences between treatments (*P* < 0.05; LSD).

Previously, we designed a plant-protective SynCom comprising four Gram-positive bacterial species isolated from field upland soils (Lee et al., 2021). Since the drench application of SynCom systemically elicited induced resistance in tomato plants, we wanted to test whether the drench application of SynCom can protect economically important crop plants from insect pests. The roots of pepper plants grown under greenhouse conditions were drenched with the SynCom suspension, and the population of aphids on the aboveground plant parts was assessed (**Figures 1A, B**). Compared with the control, drenching with 0.5 mM BTH (positive control) reduced the aphid population size by 1.8- and 2.7-fold at 7 and 14 days post infestation, respectively (**Figure 1C**). Similar to BTH, the drench application of SynCom also reduced the population of aphids on pepper plants by 1.7- and 1.6-fold at 7 and 14 days post infestation, respectively (**Figure 1C**). However, unlike BTH, the SynCom treatment did not result in a growth penalty in pepper (**Supplementary Figure 1**).

SynCom activates the signaling of defense-related phytohormones, salicylic acid (SA) and jasmonic acid (JA), in tomato plants (Lee et al., 2021). To validate the activation of SA and JA signaling by SynCom treatment in pepper plants, we analyzed the expression patterns of defense-related marker genes involved in SA and JA signaling in pepper leaves at 24 hours post aphid inoculation (**Figure 1D**). Compared with the negative control, treatment with SynCom and BTH upregulated the expression of SA signaling marker gene *CaPR1* by 2.5-fold and 2.07-fold, respectively. In addition, SynCom treatment upregulated the expression of JA biosynthesis gene *CaLOX* by 2.74-fold compared to control, but not BTH treatment. However, the expression of JA responsive gene *CaPIN2* was downregulated by SynCom treatment compared to control. Thus, this result suggests that the SynCom treatment activates induced resistance in pepper plants against aphids.

### 3.2 SynCom enhanced resistance against aphids and *Xav* in pepper under field conditions

**Figure 2:**
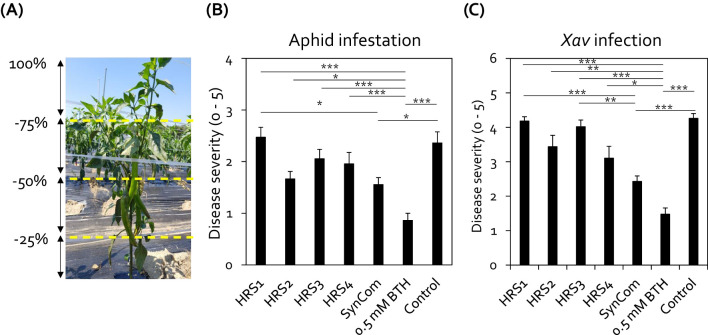
Drench application of SynCom protected pepper plants from aphid infestation and *Xanthomonas axonopodis* pv. *vesicatoria* (*Xav*) infection under field conditions. **(A)** Severity standard for aphid infestation in pepper plants in the field at Nonsan. Aphid infestation severity was recorded on a 0–5 scale, based on the proportion of aboveground tissues infested: 0 = no infestation; 1 = less than 25% of plant parts infested; 2 = 25–49% of plant parts infested; 3 = 50–74% infested; 4 = 75–99% infested; 5 = whole plant infested, resulting in plant death. **(B)** Severity of aphid infestation in pepper plants at 4 weeks after SynCom treatment. Data represent mean ± SEM of 10 plants with four block repeats (*n* = 40 replications per treatment). Asterisks indicate significant differences (*P < 0.05; **P < 0.01; ***P < 0.001; Dunn’s test). **(C)** Drench application of SynCom protected pepper plants against *Xav* infection under field conditions. Data represent mean ± SEM of five plants with three block repeats (n = 15 replicates per treatment). Asterisks indicate significant differences (*P < 0.05; **P < 0.01; ***P < 0.001; Dunn’s test). HRS1, *Brevibacterium frigoritolerans* HRS1; HRS2, *Bacillus niacini* HRS2; HRS3, *Solibacillus silvestris* HRS3; HRS4, *Bacillus luciferensis* HRS4; SynCom, mixture of HRS1–4; BTH, 0.5 mM benzothiadiazole (positive control); SDW, sterile distilled water (negative control).

To test the disease control activity of the Gram-positive bacterial SynCom under field conditions, the roots of pepper plants grown at Nonsan, South Korea, were drenched with either individual bacterial suspensions or the SynCom suspension (**Figures 2A, B**). Among the bacterial treatments, only drenching with the SynCom suspension significantly reduced aphid infestation in pepper plants by 1.5-fold compared with the negative control (**Figure 2B**). In contrast, individual application of each strain did not lead to a significant reduction in aphid infestation compared with the negative control (**Figure 2B**). Meanwhile, treatment with BTH (positive control) reduced aphid infestation by 2.7-fold compared with the negative control (**Figure 2B**).

Previously, we showed that the activation of induced resistance against both aphids and *Xav* involves the same defense signaling pathways, including SA and JA signaling (Lee et al., 2012). Thus, SynCom-mediated induced resistance led us to hypothesize that the SynCom treatment can also elicit induced resistance against *Xav* infection in pepper. Indeed, drenching with SynCom reduced the symptoms of bacterial spot disease, caused by *Xav*, on the leaves of pepper plants under field conditions (**Figure 2C**). SynCom application notably reduced the severity of bacterial leaf spot on pepper leaves by 1.4-fold compared to the negative control (**Figure 2C**). In contrast, none of the individual SynCom strains showed a significant effect on disease severity (**Figure 2C**). BTH achieved a significant 1.6-fold reduction compared to negative control, and its disease control efficacy was comparable to that of SynCom (**Figure 2C**). Taken together, these results indicate that the combination of Gram-positive bacteria (SynCom) systemically protects pepper plants from attack by insect pests and bacterial pathogens.

### 3.3 SynCom treatment increased fruit yield

**Figure 3:**
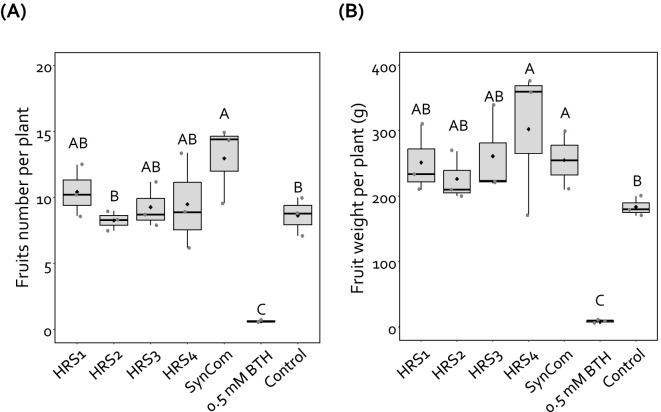
SynCom treatment increased pepper fruit yield. **(A, B)** Number **(A, B)** fresh weight of pepper fruits harvested from three blocks, each containing seven plants (*n* = 21), treated with Gram-positive bacterial species (either individually or as a mixture), BTH, and SDW. For each treatment, seven plants per block were measured and averaged, resulting in one value per block. Dots represent these block-level averages. HRS1, *Brevibacterium frigoritolerans* HRS1; HRS2, *Bacillus niacini* HRS2; HRS3, *Solibacillus silvestris* HRS3; HRS4, *Bacillus luciferensis* HRS4; SynCom, mixture of HRS1–4; BTH, 0.5 mM benzothiadiazole (positive control); SDW, sterile distilled water (negative control). Different letters indicate significant differences between treatments (*P* < 0.05; LSD). Diamonds and bolded lines of the boxplot are the average and median of indicated values, respectively.

To investigate the effect of SynCom on the fruit yield of pepper plants, we measured the weight and number of fruits harvested from SynCom-treated pepper plants in the field at Nonsan in 2019 (**Figures 3A, B**). Drenching with SynCom enhanced the fruit number and weight per plant by 1.5- and 1.7-fold, respectively, compared with the negative control (**Figures 3A, B**). The drench application of HRS4 suspension also enhanced the fruit weight per plant by 1.7-fold, but not fruit number per plant, compared with the negative control (**Figures 3A, B**). Treatment with HRS1, HRS2, and HRS3 suspensions individually did not affect pepper fruit number and weight. Meanwhile, drenching with BTH significantly reduced the pepper fruit yield (**Figures 3A, B**). Thus, SynCom treatment increased the yield of pepper fruits under field conditions.

### 3.4 *De novo* production of 1-nonanol by the SynCom deterred aphid infestation in pepper

**Figure 4:**
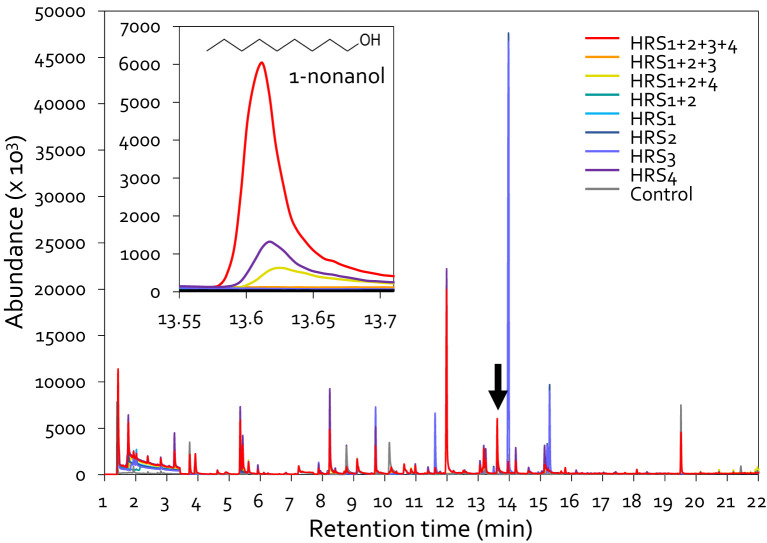
Chromatographic profiling of volatile organic compounds (VOCs) produced by Gram-positive bacteria. GC-MS of VOCs released by Gram-positive bacteria,either individually or in different combinations. The TSA medium was inoculated with bacterial suspensions in 20-mL SPME vials and incubated at 30°C for 2 days. The graph at the top left shows the peak of 1-nonanol. HRS1 + HRS2, mixture of *Brevibacterium frigoritolerans* (HRS1) and *Bacillus niacini* (HRS2); HRS1 + HRS2 + HRS3, mixture of HRS1, HRS2, and *Solibacillus silvestris* (HRS3); HRS1 + HRS2 + HRS4, mixture of HRS1, HRS2, and *Bacillus luciferensis* (HRS4); HRS1 + HRS2 + HRS3 + HRS4, mixture of all four SynCom strains. HRS1, HRS2, HRS3, and HRS4, single inoculations of SynCom strains; Control, Tryptic Soy Agar (negative control).

To identify the determinant eliciting plant immunity against aphid infestation, we analyzed the volatiles produced by SynCom bacteria by HS-SPME-GC-MS. Previously, various combinations (dual, triple, and quadruple mixes) were tested by supplementing the core strains HRS1 and HRS2 with HRS3 and/or HRS4, and the complete combination of all four strains (HRS1 + HRS2 + HRS3 + HRS4) most effectively suppressed disease development (Lee et al., 2021). Based on this, we analyzed the volatiles emitted by single strains or various combinations in which HRS1 and HRS2 were supplemented with HRS3 and/or HRS4 (**Figure 4**). We selected major compounds that were detected only in the SynCom treatment, with a match quality >90% and a peak area >1%, and were not detected in the control. 1-Butanol, 3-methyl-, Octyl chloroformate, (S)-(+)-6-Methyl-1-octanol, 1-Nonanol, and Cyclodecane were detected in SynCom (**Supplementary Table 2**, **Supplementary Figure 2**). Among five major candidates, the full SynCom (HRS1 + HRS2 + HRS3 + HRS4) treatment resulted in the production of the volatile compound 1-nonanol, with a 90% matching quality of its mass spectrum in the MS library, at levels higher than those produced in other treatments (i.e., individual or other combinatorial inoculations) (**Figure 4**). The volatile compound 1-nonanol was also detected in the HRS4 and HRS1 + 2+4 treatments, but its abundance in the SynCom treatment was 5.1- and 13.8-fold higher, respectively, at 13.6 min (**Figure 4**). Since the four bacterial strains were more effective in protecting pepper plants from aphids and *Xav* when used altogether than when used individually, we hypothesized that 1-nonanol generated by SynCom might be a key factor in reducing plant diseases.

**Figure 5:**
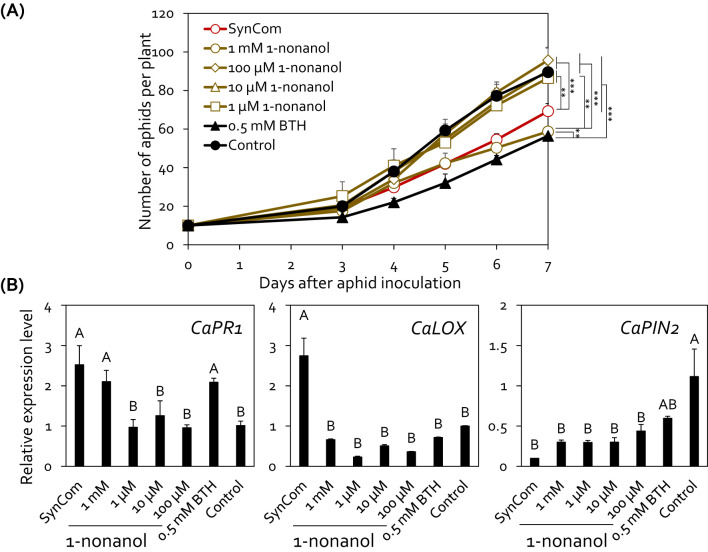
Exogenous 1-nonanol treatment protected pepper plants from aphid infestation. **(A)** Effect of 1-nonanol on aphid population size. The root system of pepper plants (*n* = 5) was drenched with 10 mL of 1-nonanol at different concentrations (1 μM, 100 μM, 10 μM, and 1 mM). The number of aphids on the aerial parts of pepper plants was counted at 3, 4, 5, 6, and 7 days post-inoculation. Comparison of treatments at different time points (Day 3 to Day 7) was performed using repeated measures ANOVA, with Group and Time as between-subjects and within-subjects factors, respectively, followed by Tukey’s honestly significant difference (HSD) *post-hoc* tests for pairwise comparisons (*p < 0.05, **p < 0.01, ***p < 0.001). The experiment was repeated three times with similar results. **(B)** Relative expression levels of salicylic acid signaling marker gene (*CaPR*) and jasmonic acid signaling marker gene (*CaLOX* and *CaPIN2*) in the leaves of pepper plants treated with 1-nonanol at 24 hour post-inoculation with aphids. Data represent mean ± SEM. Different letters indicate significant differences between treatments (*P* < 0.05; LSD). SynCom, mixture of HRS1, HRS2, HRS3, and HRS4; 1mM, 100 μM, 10 μM, and 1 μM, 1-nonanol treated pepper; BTH, 0.5mM BTH treated pepper; Control, SDW treated pepper.

To investigate the active concentration of 1-nonanol for controlling aphid infestation, we applied serially diluted concentration of 1-nonanol (1 mM, 100 μM, 10 μM, and 1 μM) to the pepper root system (**Figure 5A**). Drench application of 1 mM 1-nonanol significantly decreased the aphid population on pepper plants by 26.1%, 34.5%, 45.1%, 48.3%, and 38.9% at 3, 4, 5, 6, and 7 days post-inoculation, respectively compared with the negative control (**Figure 5A**). Significant reduction in aphid populations was also observed in the SynCom or BTH treatment at 4, 5, 6, and 7 days post-inoculation.

To investigate whether the SynCom-derived 1-nonanol activates defense signaling in pepper, we analyzed the expression patterns of defense-related marker genes involved in SA and JA signaling in pepper leaves at 24 hours post-inoculation (**Figure 5B**). Compared with the negative control, treatment with SynCom and BTH upregulated the expression of SA signaling marker gene *CaPR1* by 2.52-fold, 2.09-fold, respectively. Treatment with 1 mM 1-nonanol and BTH upregulated the expression of SA signaling marker gene *CaPR1* by 2.10-fold, compared with the negative control. SynCom treatment activated the expression of the JA biosynthesis gene *CaLOX* by 2.74-fold compared to the control, but did not affect the expression of the JA responsive gene *CaPIN2*. However, except for SynCom, the expression of the JA marker genes *CaLOX* and *CaPIN2* did not up-regulated in any 1-nonanol treatments compared to negative control (**Figure 5B**). Meanwhile, unlike BTH, exogenous 1-nonanol treatment did not result in a growth penalty in pepper (**Supplementary Figure 3**). Taken together, these results suggest that the SynCom can activate SA-dependent induced resistance in pepper plants against aphids through the *de novo* synthesis of 1-nonanol.

## 4 Discussion

Rhizosphere microbiota benefit plants by enhancing their abiotic and biotic stress tolerance. While studies have explored the interactions among beneficial microbiota, the production of unique plant-protective determinants by these microbiota remains largely unknown. Here, we demonstrate that a Gram-positive SynCom and its *de novo* synthesized metabolite, 1-nonanol, protects pepper plants from aphid infestation under greenhouse and field conditions. The SynCom showed higher biocontrol activity against aphids and *Xav* compared with individual bacterial inoculations. We found that the volatile compound 1-nonanol, produced by the combination of all four Gram-positive positive bacteria (SynCom), reduced aphid infestation in pepper plants, demonstrating how this specific bacterial combination produces a unique metabolite to enhance plant defense.

Previously, the SynCom exhibited priming effects against *Ralstonia pseudosolanacearum* SL341, which causes bacterial wilt disease in tomato (Lee et al., 2021). Consistent with this finding, when applied as a biocontrol agent in pepper, another *Solanaceae* crop, the SynCom exhibited strong disease control activity against aphids under both indoor and field conditions (**Figures 1**, **2**). In alignment with previous data, the application of all four Gram-positive bacteria (SynCom) resulted in the highest biocontrol activity compared with individual bacterium inoculations and control (**Figure 2**, Lee et al., 2021). Similar to resistance against aphids, the SynCom treatment also protected pepper plants against the semibiotrophic bacterial pathogen *Xav* (**Figure 2B**). Since the SynCom treatment primed SA- and JA-dependent induced resistance against *R. pseudosolanacearum* in tomato and aphid in pepper plant (Lee et al., 2021, **Figure 1D**), it is likely that the induced resistance triggered by the SynCom used in this study shares similarities with that induced against other phytopthogens and herbivores, including the sucking insect-pest aphid, *Xav*, and *R. pseudosolanacearum* (Walling, 2000; Kaloshian and Walling, 2005; Pieterse et al., 2009; Lee et al., 2012, 2021). In comparison with immune chemical triggers such as BTH, which can lead to excessive immune activation and growth penalties in plants, the SynCom not only enhanced disease resistance but also increased pepper fruit yield, suggesting that SynCom can systemically elicit induced resistance without growth penalties (**Figures 1**, **2**; **Supplementary Figure 1**).

Induced resistance triggered by SynCom may be a result of the production of 1-nonanol, which protects pepper plants against aphid infestation without any growth penalties (**Figures 2**, **4**, **5**, **Supplementary Figure 3**). Exogenous 1-nonanol application can systemically activate the expression of genes involved in defense phytohormone (JA and SA) signaling as well as oxidative stress responses in *Arabidopsis* and cotton plants (Gamboa-Becerra et al., 2022; Parmagnani et al., 2023; Ni et al., 2024). Consistently, treatment with either the SynCom or exogenous 1-nonanol activated the expression of SA signaling genes in pepper leaves following infestation by the sucking insect aphid (**Figures 1D**, **5B**), suggesting that SynCom-derived 1-nonanol is a key metabolite that elicits SA-dependent immunity against aphids in pepper. Interestingly, only SynCom treatment, not exogenous 1-nonanol alone, induced the expression of the JA biosynthetic gene *CaLOX* (**Figure 1D**). These indicates that in addition to 1-nonaol, SynCom-derived other metabolites might contribute to the activation of a more complex plant immunity against not only sucking insect but also broad spectrum of phytopathogens (Lee et al., 2021, **Figure 2**). Thus, while 1-nonanol serves as a key metabolite, the SynCom might activate a more complex plant immunity against broad-spectrum phytopathogens and insect pests through the combined effect of multiple metabolite(s). Traditionally, a bottom-up approach has been used to construct a plant-protective SynCom, in which the microbial characteristics of random combinations of isolated bacteria are investigated via *in vitro* tests, including enzymatic activity and metabolite production assays (Marín et al., 2021; Martins et al., 2023). However, the SynCom constructed using the bottom-up approach exhibits unstable activity in field conditions, possibly because of the complexity of microbial interactions within natural communities. A multispecies combination does not always result in additive or synergistic effects, and the presence or absence of specific taxa is crucial for determining the activity of the SynCom (Sanchez-Gorostiaga et al., 2019; Marín et al., 2021). The SynCom used in this study was constructed using a top-down approach, based on the microbiome analysis of natural rhizosphere soil (Lee et al., 2021). Among the members of our SynCom, HRS1 and HRS2 play key roles in activating plant immunity; however, the full activation of induced resistance against bacterial wilt disease requires the support of minor helper strains including HRS3 and HRS4 (Lee et al., 2021). Consistently, in this study, the complete SynCom combination (HRS1 + HRS2 + HRS3 + HRS4) showed maximum disease control activity against aphids and *Xav* in pepper plants even under field conditions (**Figures 1**, **2**). Interestingly, the emission of 1-nonanol was significantly higher in the SynCom treatment than in individual or partial combination treatments lacking HRS3 and/or HRS4 (i.e., dual or triple combinations), suggesting that minor helper strains such as HRS3 and HRS4 play a crucial role in volatile compound biosynthesis through microbial interactions within the SynCom (**Figure 4**). These results demonstrate that specific microbial combinations can maximize the production of unique metabolites, offering new evidence for microbial interdependency and syntrophic interaction (Zengler and Zaramela, 2018; Kost et al., 2023; Lee et al., 2024). Meanwhile, the 1-nonanol were also detected in single inoculation of HRS4 and in partial combinations that included HRS4, indicating that HRS4 strain is the key strain responsible for 1-nonanol production. The ability of 1-nonanol production has only been found in Gram-negative bacteria, such as *Pseudomonas aurantiaca* and *Erwinia amylovora*, and not in Gram-positive bacteria (Ni et al., 2022; Parmagnani et al., 2023). Therefore, our findings represent the first report about 1-nonanol emission from Gram-positive *Bacillus luciferensis* HRS4 and from a Gram-positive bacterial SynCom.

For decades, various microorganisms have been used individually as alternatives to synthetic pesticides. However their activity has mostly declined under field conditions, because the lack of consideration for interactions within microbial communities in nature (Lee et al., 2021; Martins et al., 2023; Lee et al., 2024). In this study, using the top-down approach, a specific combination of four Gram-positive bacteria was constructed, which demonstrated effective biocontrol activity in pepper plants not only in the greenhouse but also in the field. A specific combination of bacterial species in the SynCom can lead to the production of unique volatile compounds, in amounts greater than those produced by monocultures, to support plant health and growth. This finding provides new insights for the development of future biocontrol agents. However, further research is needed to address the following: (1) benefits provided by 1-nonanol to the SynCom in the field; (2) the mechanism of 1-nonanol-induced plant immune signaling against diverse phytopathogens and herbivores; and (3) how the SynCom-derived 1-nonanol influences native microbial communities under natural conditions. Further investigation using soil and leaf microbiome profiling could help evaluate the colonization capacity of SynCom members and their impact on native microbial communities. In addition, plant metabolomic analyses, including volatile profiling, may offer deeper insights into the immune responses activated by SynCom or 1-nonanol treatment. Although previous studies have shown that the absence of SynCom increases plant disease susceptibility, the causes of SynCom dysbiosis remain unknown (Lee et al., 2021). Preventing the dysbiosis of protective microbiota or applying their active metabolites is essential for advancing biological control strategies (Chen et al., 2020; Lee et al., 2021; Arnault et al., 2023). Future efforts should focus on increasing the abundance of protective SynCom using probiotics or utilizing SynCom-derived metabolites, such as 1-nonanol, as postbiotics for agricultural applications.

## Data Availability

The raw data supporting the conclusions of this article will be made available by the authors, without undue reservation.
