# Supplementary material for: Better than one: a synthetic community of Gram-positive bacteria protects pepper plants from aphid infestation through de novo volatile production
Source: Front Plant Sci. 2025 May 29;16:1589266. doi: 10.3389/fpls.2025.1589266 (PMC12159051; doi:10.3389/fpls.2025.1589266)
Supplement: Supplementary file 1 [file DataSheet1.docx]

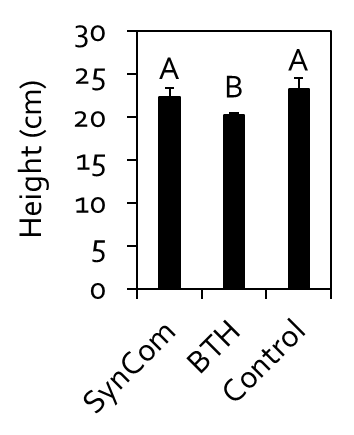

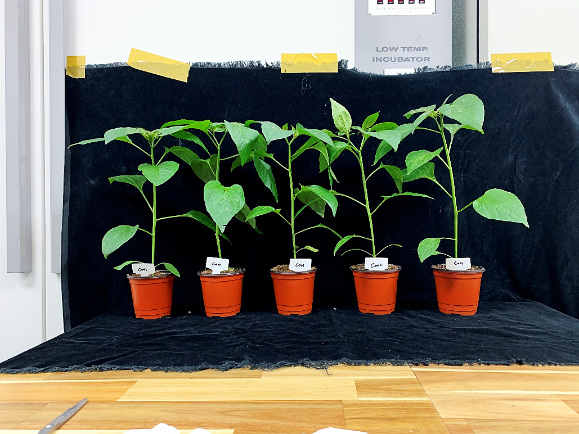

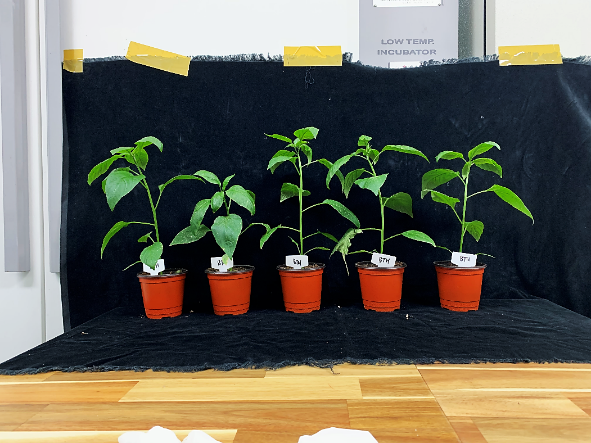

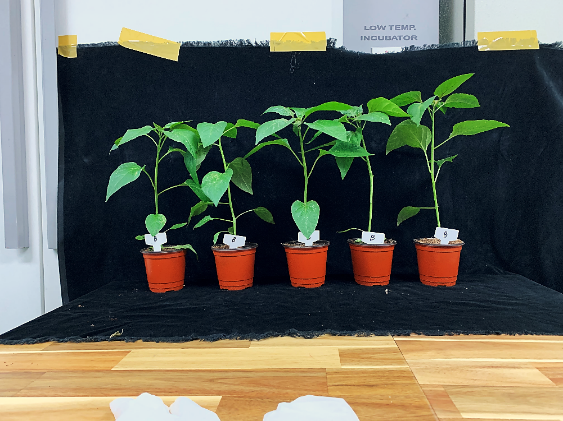


**SynCom**

**BTH**

**Control**

**(A)**

**(B)**

**Supplementary Fig. S1. Effect of SynCom on pepper growth**. (A) The photos were taken at 4 week after treatment. (B) The height of pepper plants was measured at 4 weeks post inoculation. SynCom, mixture of *Brevibacterium frigoritolerans* HRS1, *Bacillus niacini* HRS2, *Solibacillus silvestris* HRS3, and *Bacillus luciferensis* HRS4; BTH, 0.5 mM BTH; Control, SDW (negative control). Different letters indicate significant differences between treatments (*P* < 0.05; least significant difference [LSD] test, followed by Tukey-Kramer HSD post hoc test).


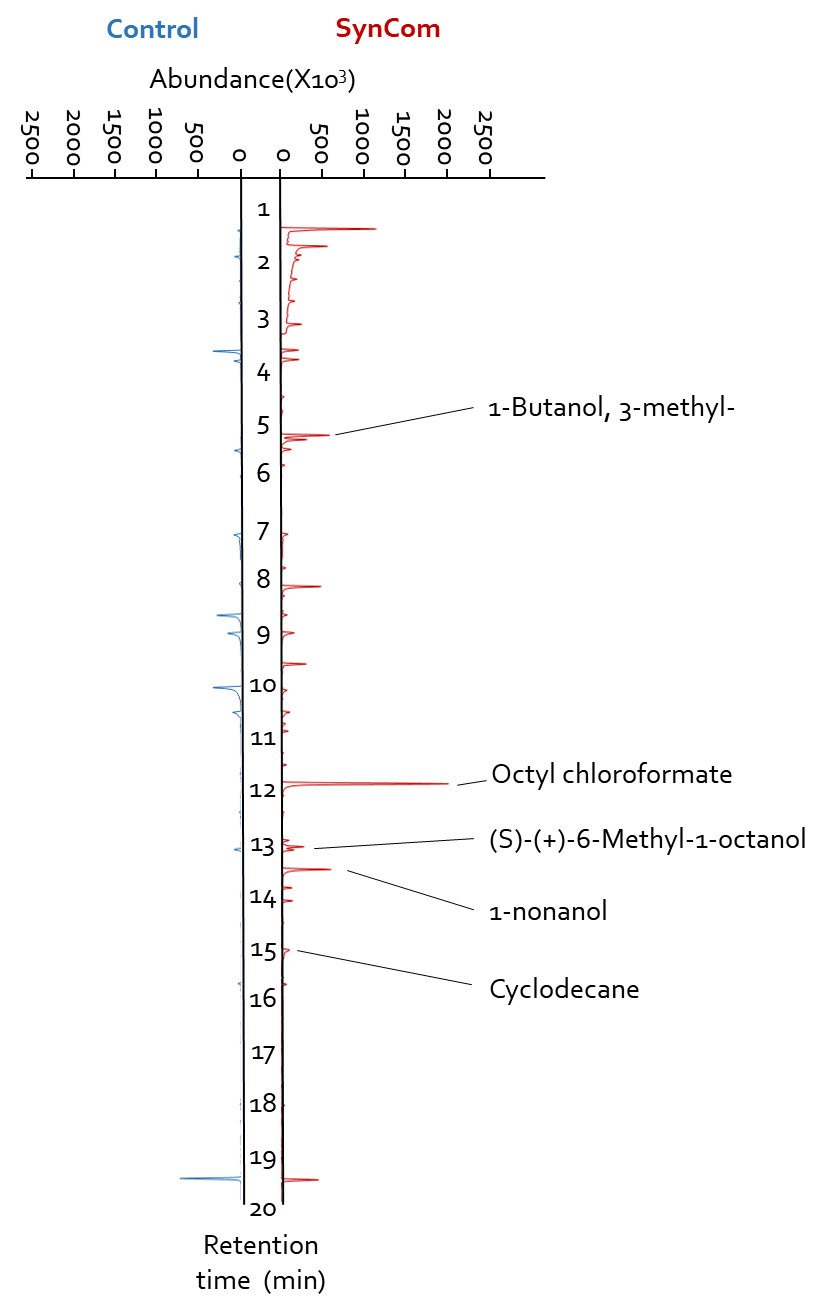


**Supplementary Fig. S2** GC-MS chromatograms of VOCs in SynCom and medium control. Five major volatile candidates emitted by SynCom compared to control. The TSA medium was inoculated with bacterial suspensions in 20-mL SPME vials and incubated at 30 °C for 2 days. SynCom, mixture of all four SynCom strains; Control, Tryptic Soy Agar (negative control).

Shoot fresh weight (g)


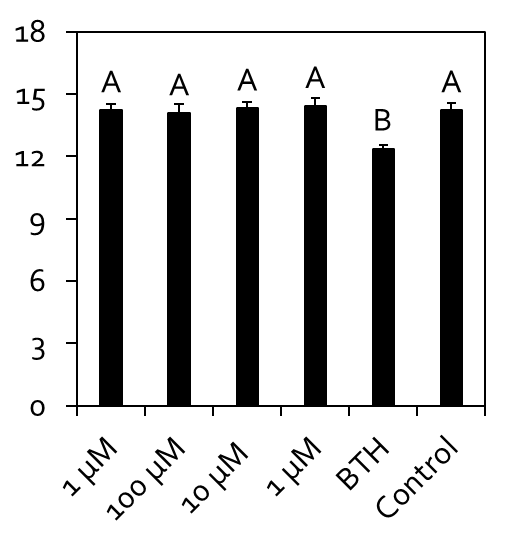


**Supplementary Fig. S3. Effect of 1-nonanol on pepper growth**. 10ml of 1-nonanl solutions (1mM, 100 μM, 10 μM, and 1 μM), 0.5mM BTH, and SDW were drenched into root system of pepper plants (*n* = 5). The fresh weight of aboveground part in pepper were measured at 5 weeks post inoculation. 1mM, 100 μM, 10 μM, and 1 μM, 1-nonanol treated pepper; BTH, 0.5mM BTH treated pepper; Control, SDW treated pepper. Data represent mean ± SEM. Different letters indicate significant differences between treatments (*P* < 0.05; least significant difference [LSD] test, followed by Tukey-Kramer HSD post hoc test). The experiment was repeated three times with similar results.

**Table S1. List of primers used for quantitative real-time PCR (qRT-PCR).**

| **Primer name** | **Primer sequences** | **Classification** |
| --- | --- | --- |
| *CaActin*-F | 5'-CACTGAAGCACCCTTGAACCC-3' | Reference gene |
| *CaActin*-R | 5'-GAGACAACACCGCCTGAATAGC-3' |  |
| *CaPR1*-F | 5'-GACATGGGACAATAGGCTAG-3' | Salicylic acid signaling marker gene |
| *CaPR1*-R | 5'-CAGTTGGAAGTTCCAATTTG-3' |  |
| *CaLOX1*-F | 5'-AATCCATTGTTCAGTTCCTTATCC-3' | Jasmonic acid signaling marker gene |
| *CaLOX1*-R | 5'-CCTCCTTCATAAAGCCTCAGT-3' |  |
| *CaPIN2*-F | 5'-TGGGACTTTCATTTGTGAAGGAGAG-3' |  |
| *CaPIN2*-R | 5'-GACACAGTGAATAGGCAATATTTGG-3' |  |

**Table S2. The list of SynCom-derived major volatile compounds selected from Fig. S2.**

| Retention time  (min) | Chemical  name | Match  quality (%) | Area  (%) | CAS  number |
| --- | --- | --- | --- | --- |
| 5.356 | 1-Butanol, 3-methyl- | 90 | 3.03 | 000123-51-3 |
| 11.986 | Octyl chloroformate | 91 | 11.01 | 007452-59-7 |
| 13.183 | (S)-(+)-6-Methyl-1-octanol | 91 | 1.62 | 110453-78-6 |
| 13.612 | 1-Nonanol | 90 | 3.99 | 000143-08-8 |
| 15.147 | Cyclodecane | 95 | 1.79 | 000293-96-9 |
